# KOFFI and Anabel 2.0—a new binding kinetics database and its integration in an open-source binding analysis software

**DOI:** 10.1093/database/baz101

**Published:** 2019-10-11

**Authors:** Leo William Norval, Stefan Daniel Krämer, Mingjie Gao, Tobias Herz, Jianyu Li, Christin Rath, Johannes Wöhrle, Stefan Günther, Günter Roth

**Affiliations:** 1 ZBSA Center for Biological Systems Analysis, Albert-Ludwigs-University Freiburg, Habsburgerstrasse 49, D-79104 Freiburg, Germany; 2 Faculty for Biology, Albert-Ludwigs-University Freiburg, Schaenzlestrasse 1, D-79104 Freiburg, Germany; 3 IMTEK Department of Microsystems Engineering, Albert-Ludwigs-University Freiburg, Georges-Köhler-Allee 103, D-79110 Freiburg, Germany; 4 BioCopy GmbH, Spechtweg 25, D-79110 Freiburg, Germany; 5 BIOSS Center for Biological Signalling Studies, Albert-Ludwigs-University Freiburg, Schänzlestrasse 18, D-79104 Freiburg, Germany; 6 Institute of Pharmaceutical Sciences, Pharmaceutical Bioinformatics, Albert-Ludwigs-University Freiburg, Hermann-Herder-Straße 9, D-79104 Freiburg, Germany

## Abstract

The kinetics of featured interactions (KOFFI) database is a novel tool and resource for binding kinetics data from biomolecular interactions. While binding kinetics data are abundant in literature, finding valuable information is a laborious task. We used text extraction methods to store binding rates (association, dissociation) as well as corresponding meta-information (e.g. methods, devices) in a novel database. To date, over 270 articles were manually curated and binding data on over 1705 interactions was collected and stored in the (KOFFI) database. Moreover, the KOFFI database application programming interface was implemented in Anabel (open-source software for the analysis of binding interactions), enabling users to directly compare their own binding data analyses with related experiments described in the database.

## Introduction

A key step in the understanding of all biological processes lies in describing the underlying interactions between biomolecules. Especially in drug discovery, characterizing binding properties of antibody–antigen, enzyme–inhibitor or receptor–ligand interactions plays a crucial role in identifying suitable drug candidates. Still, only ~14% of potential drugs make it through clinical trials (1). While drug discovery relies heavily on *in vitro* binding assays to determine binding affinity in terms of the half maximal inhibitory concentration (IC_50_) or the equilibrium dissociation constant (K_D_), this is often not directly transferable to the *in vivo* efficacy of a drug (2). Assay conditions frequently present an equilibrium state, while *in vivo* concentrations of the ligand vary over time, especially in dependency of administration way and exact formulation. This coincides with the notion that it is not the affinity of a drug itself which determines its efficacy, but the association (on-rate, k_on_) and dissociation rate (off-rate, k_off_), the latter being inversely related to the ‘residence-time’ or ‘dissociative half-life’ of the complex, as has been proposed periodically throughout the past decade (2–6). On the one hand, some biological processes require a minimum time to be accomplished, such as the activation of a G-protein coupled receptor, and thus depend on the complex to be stable for at least that period. On the other hand, for some receptor–ligand complexes where the ligand is internalized after a certain time of being bound and is subsequently degraded, a faster dissociation rate may decrease internalization and thus increase bioactivity (3). Therefore, it is apparent that the K_D_ or IC_50_ value does not provide all the information necessary to characterize the interactions, especially as the importance of the association and dissociation rates varies with different underlying biological mechanisms. Additionally, studies have shown that not all detection methods and devices yield similar binding rates for the same interaction, especially in the case of methods using labeling techniques, as they utilize either non-native proteins and/or ligands with potentially different binding properties (7,8).

With this change in the awareness and understanding of underlying processes comes an increase in demand for high-quality binding kinetics data. Several articles have been published on binding kinetics modeling and prediction of k_on_ and k_off_, using data mined from the literature (9–11). Although some databases annotating K_D_ or IC_50_ values exist (Table 1), a more general resource containing association and dissociation rates combined with crucial information on the experimental set-up is missing to our knowledge. The intent of this project was therefore to fill this gap by creating a database to collect and store binding kinetics data mainly for label-free detection methods extracted from literature with related information about the performed experiments for k_on_ and k_off_ determination, providing reference data for future experiments and high-quality data for data mining projects. It is hoped that this database will lay a foundation which can then be used for further development in the future.

**Table 1:** Comparison of information stored in binding interaction databases

Parameters	Binding MOAD	AffinDB	Ki DB	PDBbind	BindingDB	KDBI	KOFFI (This project)
Focus	PDB protein–ligand (small molecules, short peptides, oligo-nucleotides, cofactors) complexes	Protein–ligand complexes of the PDB	Drugs and drug candidates	Biomolecular complexes of the PDB	Drugs and drug candidates (including data from other databases, such as AffinDB)	All biomolecular interactions described in literature	All biomolecular interactions described in open-access literature
K_D_/K_A_	✔	✔	✕	✔	✔	✔	✔
k_on_ & k_off_	✕	✕	✕	✕	✔	✔	✔
IC_50_	✔	✔	✕	✔	✔	✔	✕
K_I_	✔	✔	✔	✔	✔	✔	✕
ΔG	✕	✔	✕	✕	✔	✕	✕
PDB Subset	✔	✔	✕	✔	✔	✕	✕
Method	✕	✔	✕	✕	✔	✕	✔
Device	✕	✕	✕	✕	✔	✕	✔
Rating	✕	✕	✕	✕	✕	✕	✔
Manual curation	✔	✔	**?**	✔	✔	✔	✔

## Results and Discussion

### Database access

The kinetics of featured interactions (KOFFI) database is currently available at www.koffidb.org. Search results are displayed in tabular form with minimal binding information and links provided to a detailed description. Additionally, all annotated interactions are available for download in CSV format. Apart from its website, it is also possible to access the database via a REST application programming interface (API).

### Similar resources

A variety of similar resources exists, some with extensive data on equilibrium constants, but without information on association or dissociation events, such as Binding MOAD (12), AffinDB (13), Ki DB (14) or PDBbind (15). Others such as BindingDB (16) and KDBI (17) provide partial data for k_on_ and k_off_. While BindingDB contains chiefly data on small drug-like compounds and has partial information on the experimental set-up, KDBI is not restricted to a particular type of interaction. Unfortunately, KDBI does not store any information on the used method, device, chip or software and generally suffers from a lack of experimental information. KOFFI provides details not only on the experimental set-up, but also contains a rating system, giving a direct measurement for the quality of the experimental data. A brief comparison is shown in Table 1.

### The binding kinetic landscape

**Figure 1:**
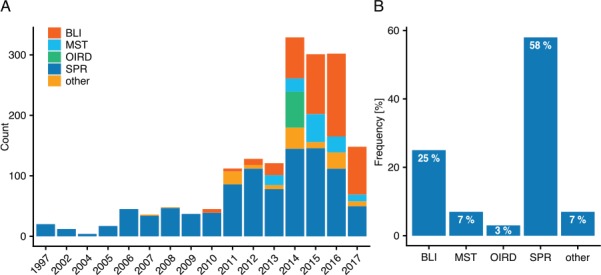
**(A)** Annotated interactions by year and method. Depicted are the yearly interaction counts for major methods in the order of appearance. **(B)** Overall interactions annotated by method (rounded).

Finding the right literature containing valuable data is no simple task—PubMed returns over 184 000 (July 2018) search results when querying for ‘binding affinity’ and on average, over 5000 articles were published annually for the past 20 years. What first comes to mind when trying to mine such literature for data is to try using automated methods for retrieving the needed information. While this may be suitable for other tasks, such an approach is more difficult for creating a dataset for binding rates. A major issue is the missing of a standardized structure of kinetic data within publications. One problem is that there seem to be no international guidelines on publishing data from binding kinetic experiments—some authors merely mention rate constants within the text, while others store them in tables which, unfortunately, are often very dissimilar, and sometimes kinetic data are only displayed within images and figures. A further issue arises due to PDF files—although the documents data are preserved, they are not saved uniformly. This makes bulk conversion of PDFs to a mineable format very error-prone, as some text may be stored as images, contain unknown fonts or have other inconsistencies across documents. Thus, the focus was set on articles from the PMC Open Access Subset, as these do not suffer from other journals license restrictions and are available in XML and text file format. Over 1.59 M PMC articles were stored and indexed, allowing custom queries to select and rank relevant articles. As the annotation process could not be automated, a web-based manual annotation tool was developed to provide users a better experience while curating the articles. Using this tool in a collaborative effort, 270 articles corresponding to 1705 individual binding events were annotated by 9 experts. The annotated binding interactions stem from more than 10 different measurement methods (Figure 1A). Overall, Surface Plasmon Resonance (SPR) takes up the biggest share with 58% of all detection methods, followed by 25% for Bio-Layer Interferometry (BLI), 7% for MicroScale Thermophoresis (MST), 3% for Oblique Incidence Reflectivity Difference and a total of 7% for all other methods (Figure 1B). It is noteworthy that this statistic is based on a small fraction of the entire binding data available and that some methods such as Isothermal Titration Calorimetry may be under-represented due to a non-representative selection of literature. However, it does provide a rough estimate of how great a foothold these methods have gained within the scientific community.

Focusing on the major methods, an interesting development can be seen over the years (Figure 1A). The first commercial SPR biosensors were developed in 1990 (18), whereas as BLI emerged as a new technology with ForteBio releasing their Octet System in 2005 and later other technologies such as MST catching hold. In Figure 1A both SPR and BLI appear several years later than their original technology release, which may be both because of the time it took for first experiments to be successfully published, the limited number of articles annotated in this project or the restriction to open-access articles.

**Figure 2:**
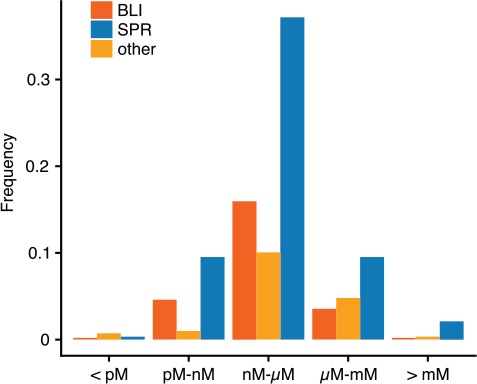
Normalized distribution of KD values by method. Shown are the frequencies of interactions with a KD in a specific range for the major methods SPR and BLI. Other methods are summarized.

The influence of this history of binding experiments can also be seen in Figure 2, where K_D_ values detected by SPR are distributed in a slightly higher range in comparison to BLI-detected dissociation constants. This reflects the earlier impact of SPR on binding experiments, as detection limits were notably higher during earlier stages of the technology.

### Data quality

**Figure 3:**
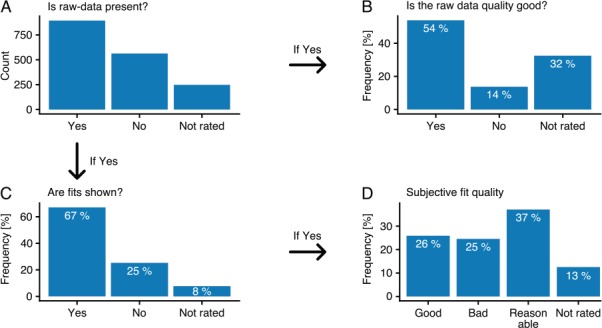
Data quality. All annotators were encouraged to rate the underlying data of the binding events using four different questions **(A–C)**. Hereby, the graphs B and C are relative to data classified with ‘Yes’ in graph A. Graph D shows the relative values to the data classified with ‘Yes’ in graph C.

As stated previously, data are represented in quite varying and inconsistent ways throughout the publications. Not only are results shown in differently structured tables, but the quality of the raw data and fits, if shown at all, varies to a great degree. Since the articles needed to be manually annotated in any case, an additional rating section was added to each interaction during annotation. The rating section was comprised of four simple questions, concerning presence and quality of raw data and of fitted curves (where present), that annotators were encouraged to answer. Over 33% percent of interactions did not have any associated raw data present in the respective article, and where it was present, only 54% could clearly be classified as good quality raw data (Figure 3A and B). Even in cases where raw data were present, fits are only shown in 67% of the cases. Where present, fits were classified as good in only about 26% of all cases, with most being classified as reasonable (37%) and 25% classified as bad. It is important to note that the category ‘not rated’ includes fits and raw data that annotators could not undoubtingly classify into the other categories (e.g. when raw data were not clearly labeled or the fitting quality was in between neighboring categories), so that the statistic may be biased toward interactions where raw data and fits do not clearly fit within the defined categories.

**Figure 4:**
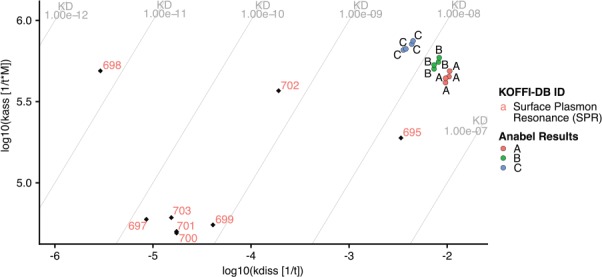
kdiss/kass plot produced using Anabel’s ‘KOFFI database analysis’ module. The evaluated real-life dataset is illustrated as colored dots, whereas the database search results are shown as colored labels with black rhombuses. KD lines are drawn as gray lines with their corresponding values at the edges of the graph.

Although annotators were advised on how to classify fits into ‘good’, ‘reasonable’ and ‘bad’ categories, it was impossible to prevent the ratings from suffering from a certain degree of subjectivity. To illustrate this point, three interactions were randomly selected from each rating category (Supplement S1).

### Anabel integration

Anabel is an online tool for the analysis of biomolecular binding events (19). It is open source, accessible online (www.anabel-online.com) and can handle data from multiple resources. With the development of the KOFFI database, we extended Anabel with a module called ‘KOFFI database analysis’. Here, users can directly search the KOFFI database within Anabel and compare the search results with their own binding data analyses. Anabel queries the KOFFI database API and subsequently generates an interactive k_off_/k_on_ plot illustrating the selected data points from both the database and their own analysis. To illustrate the full potential of Anabel, a single curve analysis of Anabels supplied real-life dataset was performed and its results compared with thrombin aptamer interaction measurements from the database (Figure 4). The database was searched for the core aptamer sequence (GGTTGGTGTGGTTGG) used in the example dataset and all database search results containing this core sequence were included. All selected data points from the database were plotted using their unique identifier. Data points from the Anabel analysis are illustrated as colored points. The obtained k_off_ values are much larger than most of the ones found in the database, whereas the k_on_ values are in a similar range to about half of the database values. Subsequently, the calculated K_D_ values of the performed analysis tend to be larger by several orders of magnitude than the values found in the literature. One possible reason could be that all of the database points originate from SPR measurements, yet the analyzed real-life dataset originated from a BLI measurement. Using the supplied database unique identifier, it would now be possible to further compare the differences of measurements by having a look into the corresponding paper. At the end of each ‘KOFFI database analysis’ it is possible to download an excel file containing all the necessary information as well as the detailed results from the performed database search.

### Outlook and maintenance

The main goal during development of the KOFFI database was to create a resource for high-quality binding kinetics data for use in a variety of analyses including the creation of training and test datasets for kinetic modeling and prediction methods or finding systematic tendencies between methods, devices and interaction types. Moreover, the Anabel KOFFI module integration enables straight forward comparisons of users binding kinetic experiments with the collected datasets. As such, we hope Anabel and the KOFFI database provide a solid foundation for the discussion and interpretation of future binding kinetic experiments.

Henceforth, we will provide regular database updates to further increase the number of available binding interactions. Additionally, users can send in their own annotations using the annotation excel file as provided on the KOFFI website. All external input will still be subject to scrutiny from expert curators of the database. While manual curation provides a level of quality that automated methods cannot supply, it is a very time-consuming process. As briefly mentioned earlier, current literature does not describe kinetics data in a uniform, structured manner, thus making automated preprocessing steps hardly a viable solution for the present. However, future publications will hopefully have guidelines covering publication of kinetics data in structured format. As several of such guidelines exist for other types of data, similar criteria should be defined for binding kinetics data as well. A short example of what a possible set of such rules for publishing binding kinetics data could look like, has been added in the Supplementary Information (Supplement S2) of this article. A uniform way of describing the data in the literature will have several positive effects. For one, the data become more easily accessible for the readers and software. But more importantly, such a guideline ensures that published data are always complete. We hope that the KOFFI database will serve as a starting point to standardize the representation of binding kinetic data in the future.

## Methods

### Article selection

The NCBIs Open-Access subset of PMC (PubMed Central) articles, containing approximately 1.6 M articles available under the Creative Commons license, was retrieved using their FTP-service. All articles were downloaded as plaintext and indexed using xapian (V.: 1.3.4). An initial query resulted in 30 509 hits, but despite the restrictions there were many high-ranking articles that did not contain any binding data.

To specify the query, the first 20 articles were categorized into relevant (containing binding data) and irrelevant (containing no binding data) articles. Using this set of relevant articles, additional terms using xapians Rset (relevance Set) and Eset (expand Set) classes were defined. These terms served as a guideline for adjusting the query with a different weighting resulting in a new ranking within the selected hits. Top ranking articles were chosen for manual annotation, for which individual article packages containing the article in XML format and other material such as pictures, tables and supplementary files were downloaded from the NCBIs FTP-Service.

### Data structure

Articles were manually curated using self-developed annotation tool. The annotation tool was written using the Django web-framework (V.: 2.0.1) with a PostgreSQL (V.: 9.6.9) backend. Articles were converted from XML to HTML using XML Calabash (V.: 1.1.16–97) with JATS preview XSLT stylesheets and served via the Django framework. Documents and the corresponding interactions were stored in separate tables in a PostgreSQL database using two Django model classes.

### Data annotation and curation

Documents were displayed in list form on the landing page with links referring to each articles annotation view. A permission system allowed only one user to edit an article at a time. In the annotation view users could enter information into predefined fields in a form (Supplement S3) while simultaneously viewing the article on the same page. To ensure a high quality, the annotated data were placed under additional scrutiny. Annotators were encouraged to rate the quality of binding curves and their corresponding fits, if shown (Supplement S3, rightmost column).

### Data availability

The KOFFI database can be downloaded on the KOFFI database website (www.koffidb.org). Anabel is available as an online tool (https://skscience.org/anabel, www.anabel-online.com or anabel.skscience.org) and on github (https://github.com/SKscience/Anabel).

## Authors’ contributions

G.R., S.G., L.W.N. and S.D.K. jointly developed the initial idea. L.W.N. wrote the first draft of the manuscript. L.W.N. designed and set up the annotation tool as well as the official Django server. L.W.N., S.D.K., M.G., T.H., J.L., C.R., J.W. and G.R. jointly annotated most of the scientific articles. S.D.K. designed and set up the implementation of the KOFFI-DB into the Anabel software. L.W.N. and S.D.K. analyzed the data. S.D.K., G.R. and S.G. contributed to the writing of the manuscript. G.R. and S.G. agreed with manuscript results and conclusions. All authors made critical revisions and approved the final version. All authors reviewed and approved the final manuscript.

## Supplementary Material

Supplement_revision_baz101

## Notes

**Database URL:** www.koffidb.org
