# Supplementary material for: KOFFI and Anabel 2.0—a new binding kinetics database and its integration in an open-source binding analysis software
Source: Database (Oxford). 2019 Oct 11;2019:baz101. doi: 10.1093/database/baz101 (PMC6790968; doi:10.1093/database/baz101)
Supplement: Supplement_revision_baz101 [file supplement_revision_baz101.docx]

**S1: Examples for fits rated by annotators as “Bad” (A), “Reasonable” (B) and “Good” (C).**

The neighboring categories clearly show an overlap (e.g. supplement S1 Ai with Biii or Bii with Ci) while the categories “bad” and “good” are clearly distinct from each other. Individually, while some curves rating may fit into a neighboring category, overall the ratings appear to reflect the actual quality of the data. Thus, the rating provides an indication of the data’s quality. Overall the majority of publications do not directly include raw data or fits to their data, and when they do, their quality is more often bad rather than good. Similar conclusions have been made by Rich & Myszka in their yearly surveys of biosensor literature(1–9).

**A** **i)** Interaction of mAB A20G2 and vaccinia virus protein A33.(10) Method: BLI. **ii)** Binding of the lectin HOL-18 to Porcine Stomach Mucin (PSM).(11) Method: SPR. **iii)** Binding of VEGF_165_-ATTO 647N to CD44s.(12) Method: Fluorescence Correlation Microscopy (FCS).
**B** **i)** Interaction of Grx5 with Ssq1.(13) Method: MST. **ii)** Binding of RNA-aptamer CLN64 to c-Met.(14) Method: BLI. **iii)** Binding of SecB to MBP.(15) Method: BLI.
**C** **i)** Binding of gp42 C114S to EBV gHgL.(16) Method: BLI. **ii)** Binding of vWC2-3 to Tsg.(17) Method: SPR. **iii)** Binding of the chemical compound 991 to AMPK complex α1β1γ1.(18) Method: BLI.
Adapted from cited references under the Creative Commons Attribution license.

**S2: Important aspects of kinetic data submission**

As previously stated, information on binding interactions is shared varyingly throughout publications. Guidelines for publishing data from different methods in biosciences do exist (such as the Minimal Information About a Microarray Experiment (MIAME) guideline), but to date none cover information on publishing data from binding interaction experiments. Below is a brief overview of current issues and their possible solutions:

Key points for kinetics data submission:

1. **Interaction Partners with unique identifiers**:
   While many publications present the interacting entities, not all do so in a comprehensible manner.
   **Proposal:** Each entity should be displayed in a separate column and clearly labeled as interaction partner, i.e. interaction partner 1/2.
   **Proposal:** Columns should not be subdivided. Additional classifications such as Igg vs. Fab must be derivable from the identifying column or get an entry in an additional column.
2. **Binding parameters**:

Currently, several formats are used to display rate constants, such as different SI-prefixes (e.g. mM), units within the table row or sub-multiples (e.g. 10^4^) displayed as a separate factor in the header.
**Proposal:** Only use non-prefixed units (e.g. [M]) in the header and display sub-multiples in the corresponding cells in scientific notation (e.g. 1.9e-9)
**Proposal:** Display standard deviation in separate columns next to their corresponding value.

1. **Method and Device used**
   Currently methods and devices are only mentioned in the method-section of the corresponding article. But both influence the result in varying manner and should be directly visible alongside the data, especially in publications where multiple methods have been used.
   **Proposal:** Display both in separate columns.

**S3: Form fields shown in the annotation view.**

Shown are fieldnames (black), additional descriptions/units (green) and examples for each field (blue). The rightmost column shows the rating section, comprised of four questions regarding the quality of raw data and corresponding fits.

**References:**

1. Rich, R. L. and Myszka, D. G. (2008) Survey of the year 2007 commercial optical biosensor literature, *J. Mol. Recognit.*, **21**, 355–400.

2. Rich, R. L. and Myszka, D. G. (2007) Survey of the year 2006 commercial optical biosensor literature, *J. Mol. Recognit.*, **20**, 300–366.

3. Rich, R. L. and Myszka, D. G. (2006) Survey of the year 2005 commercial optical biosensor literature, *J. Mol. Recognit.*, **19**, 478–534.

4. Rich, R. L. and Myszka, D. G. (2005) Survey of the year 2003 commercial optical biosensor literature, *J. Mol. Recognit.*, **18**, 1–39.

5. Rich, R. L. and Myszka, D. G. (2003) A survey of the year 2002 commercial optical biosensor literature, *J. Mol. Recognit.*, **16**, 351–382.

6. Rich, R. L. and Myszka, D. G. Survey of the year 2007 commercial optical biosensor literature. *J. Mol. Recognit.* **2008**, *21*, 355–400.

7. Rich, R. L. and Myszka, D. G. (2000) Advances in surface plasmon resonance biosensor analysis, *Curr. Opin. Biotechnol.*, **11**, 54–61.

8. Rich, R. L. and Myszka, D. G. (2000) Survey of the 1999 surface plasmon resonance biosensor literature, *J. Mol. Recognit.*, **13**, 388–407.

9. Rich, R. L. and Myszka, D. G. (2010) Grading the commercial optical biosensor literature - Class of 2008: “The Mighty Binders,” *J. Mol. Recognit.*, **23**, 1–64.

10. Matho, M. H., Schlossman, A., Meng, X., et al. (2015) Structural and Functional Characterization of Anti-A33 Antibodies Reveal a Potent Cross-Species Orthopoxviruses Neutralizer, *PLOS Pathog.*, **11**, e1005148.

11. Matsumoto, R., Fujii, Y., Kawsar, S. M. A., et al. (2012) Cytotoxicity and Glycan-Binding Properties of an 18 kDa Lectin Isolated from the Marine Sponge Halichondria okadai, *Toxins (Basel).*, **4**, 323–338.

12. Volz, Y., Koschut, D., Matzke-Ogi, A., et al. (2015) Direct binding of hepatocyte growth factor and vascular endothelial growth factor to CD44v6, *Biosci. Rep.*, **35**, e00236–e00236.

13. Uzarska, M. A., Dutkiewicz, R., Freibert, S.-A., et al. (2013) The mitochondrial Hsp70 chaperone Ssq1 facilitates Fe/S cluster transfer from Isu1 to Grx5 by complex formation, *Mol. Biol. Cell*, **24**, 1830–1841.

14. Piater, B., Doerner, A., Guenther, R., et al. (2015) Aptamers Binding to c-Met Inhibiting Tumor Cell Migration, *PLoS One*, **10**, e0142412.

15. Huang, C., Rossi, P., Saio, T., et al. (2016) Structural basis for the antifolding activity of a molecular chaperone, *Nature*, **537**, 202–206.

16. Sathiyamoorthy, K., Jiang, J., Hu, Y. X., et al. (2014) Assembly and Architecture of the EBV B Cell Entry Triggering Complex, *PLoS Pathog.*, **10**, e1004309.

17. Troilo, H., Barrett, A. L., Zuk, A. V., et al. (2016) Structural characterization of twisted gastrulation provides insights into opposing functions on the BMP signalling pathway, *Matrix Biol.*, **55**, 49–62.

18. Xiao, B., Sanders, M. J., Carmena, D., et al. (2013) Structural basis of AMPK regulation by small molecule activators., *Nat. Commun.*, **4**, 3017.
